# Literacy for Frailty among Undergraduate Medical Education: An Under-Recognized Opportunity to Improve Geriatric Care

**DOI:** 10.14336/AD.2023.0925

**Published:** 2024-08-01

**Authors:** Chia-Ter Chao

**Affiliations:** ^1^Nephrology division, Department of Internal Medicine, National Taiwan University Hospital, Taipei, Taiwan.; ^2^Nephrology division, Department of Internal Medicine, National Taiwan University College of Medicine, Taipei, Taiwan.; ^3^Graduate Institute of Toxicology, National Taiwan University College of Medicine, Taipei, Taiwan.; ^4^Center of Faculty Development, National Taiwan University College of Medicine, Taipei, Taiwan.

**Keywords:** frailty, geriatrics, health literacy, undergraduate education

## Abstract

Our society is aging much faster than it was before, and this phenomenon demands concerted action to optimize geriatric care. Presentations, clinical features, and management decision making are distinct between older adults and the general population, and to enhance care quality, there remains unmet needs for undergraduate geriatric education. Among all geriatric syndromes that clinically matter, frailty is particularly instrumental, serving as the overarching phenotype that connects other geriatric conditions and predisposes individuals to adverse outcomes. However, understandings for frailty, or “literacy for frailty” is often poor among healthcare professionals, and misidentification, terminology confusion, and uncertainty surrounding the care of frail older adults, are not uncommon. This lack of frailty literacy undoubtedly contributes to the suboptimal geriatric care patients receive. We therefore propose a rationally designed, concise, and structured program for eliciting medical students’ motivation for understanding frailty during their undergraduate period. Our increasing-frailty-literacy program includes 7 modules, accommodating the terminology, integrative pathogenesis, epidemiology of frailty, appropriate screening and identification tool selection, prognostication and patient communication, and individualization of treatment strategies. In combination with digital technologies and hands-on practice opportunities, we believe that our curriculum can promote medical students’ learning efficacy for frailty and improve geriatric care for the current generation.

## An incoming era of aging society: how to improve geriatric care?

It is widely recognized that our population is getting older than it was decades ago; epidemiologists estimate that up to 14% of global population will be older than 65 years in 2040 [1]. This “demographic transition” results from the combination of better medical care and public health measures, fertility changes, and baby booming coupled with the effect of longevity achievement [2]. Older adults are at a heightened risk of complications, resulting from multimorbidity, polypharmacy [3], functional and/or psychosocial deterioration [4]. These issues intertwine, and their interplay underlies the difficulty in providing optimal geriatric care during the contemporary era. Symptomatology is also complex in this population, and treatments directed toward symptom relief frequently churn outside effects despite the insufficient efficacy obtained. Bodily impairment precludes older adults’ capacity to live their life fully, and caregiver distress emerges following the extended period of accompanying and daily activity assistance [5]. Ethical dilemma frequently appears during geriatric care and necessitates constant reflection on how we can improve our treatment strategies.

Older adults are further inclined to developing geriatric syndromes (GS), a plethora of pathologic phenotypes constituting the unique features of aging and carrying adverse prognostic influences [6]. Relevant GS examples include delirium, incontinence, falls, sarcopenia, frailty, etc. that garner widespread attention from clinicians and researchers aiming to promote healthy aging. Among the spectrum of GS, frailty is particularly noteworthy, and has been pinpointed as the overarching phenotype that co-exists and predisposes older adults to other geriatric conditions [6]. In the following section, we will introduce the concept of frailty, literacy for frailty, and the necessity of integrating frailty into undergraduate medical education.

## What is frailty and why is it important but under-appreciated? Literacy for frailty

Frailty characterizes an individual’s susceptibility to harsh insults and arises from cumulative subtle deficits over chronologic and/or biologic aging [7]. The incidence of frailty is estimated at 43.4 cases per 1,000 person-year among older adults, and its prodrome, pre-frailty, occurs at 150.6 per 1,000 person-year [8]. Risk factors for frailty development in older adults and other diseased populations include advanced age, economic disadvantaged groups, those with poor physical performance, cognitive and balance issues, sensory dysfunction, suboptimal mental well-being, etc. [9,10]. Pathogenesis of frailty has evolved considerably over time, from the synergistic effects of multi-dimensional subclinical defects to a new conceptual model of homeostatic disturbance/instability between multiple dimensions including genetic, biological, psychologic, cognitive, and functional ones [11]. Prognostic importance of frailty can be inferred from its intimate relationship with the risk of mortality, disability, and individual organ deterioration [6,12]. Frailty also increases healthcare resource consumption and contributes to rising medical costs in various populations [13], whereas treatment decisions may also be influenced by frailty [14].

In Taiwan, the prevalence of frailty ranges between 5% and 30% among community-dwelling older adults according to prior studies [15,16], but the routine assessment for frailty in local at-risk population remains uncommon. Despite extensive clinical research addressing frailty, front-line clinicians frequently fail to proactively identify and manage frailty during primary care. A majority of healthcare professionals have limited pre-existing knowledge of what frailty means, confusing frailty with ageing or disability whereas unable to acknowledge its uniqueness and importance [17]. Even those who can identify frailty in primary care have low confidence in affirming such diagnosis, often informal and opportunistic [18]. We propose that this lack of understanding for frailty be termed poor “literacy for frailty” among healthcare professionals. This phenomenon is pivotal in that un-identified frailty compounds the adverse impact of aging on current healthcare system, rendering healthy ageing unattainable and prevention strategies futile. Contributors to poor literacy for frailty among clinicians are largely attributable to poor education and training [17,19]. Consequently, targeting undergraduate medical education to enhance such literacy becomes an attractive approach.

## Geriatric education during the undergraduate phase: putting frailty into context

Education for geriatric medicine remains at its infancy in multiple countries [20], in stark contrast to the population ageing and the rising need of appropriately trained geriatrician workforce. Moreover, it is gradually appreciated that clinicians, not just geriatricians, should be trained for the provision of optimal care for older adults. However, a prior survey in European countries showed that great heterogeneity existed in geriatric training among undergraduate education, requiring consistent effort for reinforcement and harmonization [21].

Similarly, although several countries have incorporated frailty into postgraduate and undergraduate curricula, frailty education remains poorly defined and challenging to implement. Educators are unfamiliar with the biological meaning, construct, and dimensionality, and how to assess frailty with insufficient clinical experiences; students tend to see this syndrome negatively or describe it casually [22]. Therefore, we need a more concise, structured, and rational program for eliciting medical students’ motivation for understanding frailty during their undergraduate period.

### A potential program for increasing undergraduate “frailty literacy”

**Figure 1.**
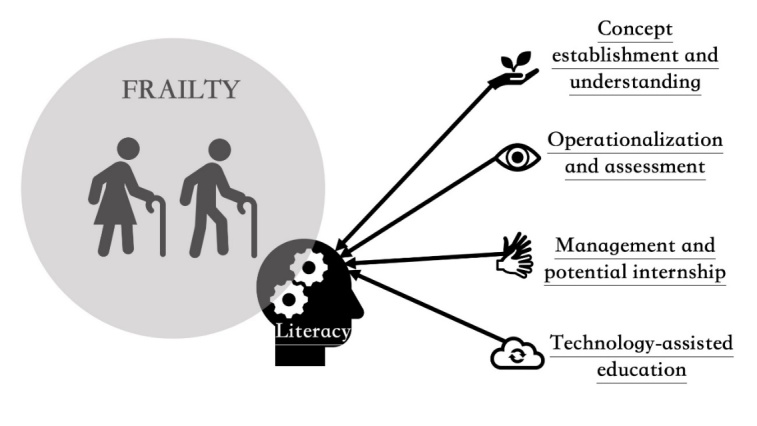
How to increase “frailty literacy” in undergraduate education: a graphical illustration.

A module aiming at acquainting medical students with the meaning and clinical aspects related to frailty is urgently required. We name the understanding of frailty-related facts/findings as “frailty literacy” in order to raise its awareness, named after the concept of “health literacy”. Health literacy refers to the degree to which one possesses the will and ability to understand and use information, materials, and services to meet his/her health demands, as defined by the Healthy People 2030 initiative (https://health.gov/healthypeople/priority-areas/health-literacy-healthy-people-2030). We therefore extrapolate this construct to frailty, aiming to promote medical students’ motivation to learn frailty and to apply relevant construct to their future practice. A putative illustration of our intent is provided in Figure 1.

We propose the following program to achieve this goal (Table 1). The program can be divided into 7 modules, accommodating the background of how frailty is coined and applied to clinical medicine, prior attempts at disentangling frailty pathogenesis, outlining the epidemiology of frailty, how to select an appropriate tool for screening and identifying frailty, understanding its prognostic influences to facilitate patient communication, and existing treatment strategies. Didactic lectures will be the main context in the three modules, and we also include hand-on opportunities in the operationalization, outcome, and management parts with real-world cases (Table 1), in order to invigorate students’ motivation and dedication. Part of the modules can also be delivered online, according to our prior experiences in small group tutorial curricula involving basic/clinical medical sciences [23,24].

**Table 1:** Our proposed core modules for increasing undergraduate “frailty literacy”.

Number	Module title	Description
**1**	History, Terminology & Inception of Frailty in Clinical Medicine	Frailty evolves from a literal description of individual’s weak state to a medical term. Students are guided through the path of the medicalization history of frailty and how this term changed over the past 2 decades. Students are expected to be able to clarify the terminology after the course.
**2**	Biologic Meanings and Evolving Pathophysiologic Understandings of Frailty	The origin of frailty is constantly under active investigation. Biomarker studies and experimental animal models of frailty have offered much insight into its role in aging biology. Students will be offered pathophysiology-oriented instruction of how frailty occurs from cell to organism level, and what frailty symbolizes in clinical medicine.
**3**	Epidemiology of Frailty in Various Affected Populations	Frailty not only occurs in older adults but also secondarily in individuals with different illnesses. We will outline the incidence, prevalence, ethnic and socioeconomic influences, and disease-specific risk factors for frailty. Students are expected to be aware of how many patients in their community may have frailty and what clinical features place them at risk.
**4**	Operationalization of Frailty Concept: Assessment Tools and Advantages/Disadvantages	The appropriate tools for assessing frailty can differ according to surveyed populations, resources, and practice convenience. Each tool has its pros and cons, and students are expected to acquaint themselves with at least 1 tool they prefer. They should also understand the drawbacks of their tool.
**5**	Functional and Overall Outcome Relevance of Frailty	Frailty significantly increases the risk of a diverse spectrum of complications, apart from higher mortality or disability. Students are expected to understand and name at least 3 frailty-related complications, so that they can communicate the necessity of frailty assessment to older adults they care for. Students can tabulate their knowledge and are encouraged to create their own toolkit for knowledge dissemination.
**6**	Optimal Management of Frailty: Optimizing Geriatric Care	Management for frailty constitutes exercise and nutritional regimens, supplemented by disease-specific care and morbidity amelioration. After curriculum, students are encouraged to make an individualized prescription for a given frail older adult, considering the capacity/resource/preference of that adult.
**7**	Clinical practice - Frailty Internship	A final wrap-up module, consisting of out-patient clinic rotation, to increase students’ clinical skills and enhance their subsequent sensitivity to frailty phenotype.

## Challenges and future perspectives

Although several major geriatric societies support the inclusion of frailty in medical education, arguments have been raised regarding how best to implement programs aiming to enhance frailty awareness in undergraduate medication [25]. Misidentification and terminology confusion, uncertainty surrounding the care of frail older adults, and clinical decisions altered by the recognition of frailty, have all been implicated as potential factors responsible for the observed difficulty in formulating a formal targeted curriculum [22]. Notwithstanding these concerns, we design a potentially applicable undergraduate program with practial modules and course descriptions to enhance frailty literacy. Our proposed program further benefits from the admixture of lectures and hands-on practices, followed by clinical encounters to deepen students’ understanding of frailty. However, compulsory undergraduate education often fails to achieve curricular goal in Asian countries [26], whereas course quality and student motivation promotion can be key to increase student uptake and improve learning efficacy. Our design may obviate the above limitations partially. It is worth noting that our curricular design should be tested in a dedicated educational setting before wider application. We plan to add more active learning elements into this program, and preferably embed this module into the course of undergraduate geriatric medicine curriculum. Technology-assisted education, including computer-based platform, simulation, virtual or augmented reality-based, internet-based synchronous or asynchronous delivery, can potentially be more helpful [27]. We hope to successfully improve geriatric care for the future generation of clinicians caring for older adults.
